# Tailoring a Low Young Modulus for a Beta Titanium Alloy by Combining Severe Plastic Deformation with Solution Treatment

**DOI:** 10.3390/ma14133467

**Published:** 2021-06-22

**Authors:** Anna Nocivin, Doina Raducanu, Bogdan Vasile, Corneliu Trisca-Rusu, Elisabeta Mirela Cojocaru, Alexandru Dan, Raluca Irimescu, Vasile Danut Cojocaru

**Affiliations:** 1 Department of Industrial Management, Ovidius University of Constanța, 900527 Constanța, Romania; anocivin@univ-ovidius.ro; 2 Department of Metallic Materials Processing and Ecometallurgy, University Politehnica of Bucharest, 060042 Bucharest, Romania; doina.raducanu@mdef.pub.ro (D.R.); bogdan.vasile@upb.ro (B.V.); mirela.cojocaru@mdef.pub.ro (E.M.C.); alexandru.dan0806@upb.ro (A.D.); raluca.irimescu@stud.sim.upb.ro (R.I.); 3 Department of Science, National Institute for Research and Development in Micro-Technologies, 077190 Bucharest, Romania; corneliu.trisca@nano-link.net

**Keywords:** β-titanium alloys, XRD, SEM, HRTEM, mechanical properties

## Abstract

The present paper analyzed the microstructural characteristics and the mechanical properties of a Ti–Nb–Zr–Fe–O alloy of β-Ti type obtained by combining severe plastic deformation (SPD), for which the total reduction was of ε_tot_ = 90%, with two variants of super-transus solution treatment (ST). The objective was to obtain a low Young’s modulus with sufficient high strength in purpose to use the alloy as a biomaterial for orthopedic implants. The microstructure analysis was conducted through X-ray diffraction (XRD), scanning electron microscopy (SEM), and high-resolution transmission electron microscopy (HRTEM) investigations. The analyzed mechanical properties reveal promising values for yield strength (YS) and ultimate tensile strength (UTS) of about 770 and 1100 MPa, respectively, with a low value of Young’s modulus of about 48–49 GPa. The conclusion is that satisfactory mechanical properties for this type of alloy can be obtained if considering a proper combination of SPD + ST parameters and a suitable content of β-stabilizing alloying elements, especially the Zr/Nb ratio.

## 1. Introduction

For more than a decade, the metastable β-titanium alloys have been considered the most promising biomaterials for load bearing implants manufacturing due to low Young’s modulus, high strength, and good ductility, a combination that can assure a suitable processability [1,2,3,4,5]. To achieve the right level of properties, there are some important requirements that must be met. One of the requirements refers to the selection of β stabilizing alloying elements. Aside from the β-stabilizing character, the biocompatibility function of these elements is very important in addition to that of titanium. From this perspective, Nb, Zr, and Ta are considered able to be used without limit due to their non-cytotoxicity character [6,7]. On the other hand, Mo and Fe can be used to a limited extent [6,8,9]. The most appreciated is Nb, which also has the strongest β-stabilizing character and high biocompatibility [1,2,10].

Another important requirement refers to the reduced costs, which imply not only a proper fabrication method with a reduced buy-to-fly ratio, but also a reduced content of expensive chemical components as Ta is. From this point of view, Fe is a good alternative being more accessible [8,9,10,11]. Therefore, many studies have tried to replace expensive Ta with Fe also considering that Ta has a very high melting temperature (T_top_^Ta^ = 2996 °C) comparative to the above enumerated, making the alloy synthesis and homogenization difficult [6,8,9,11,12].

The next demanding condition for the β-Ti alloy design refers to obtaining the minimum possible elastic modulus to avoid the so-named “stress shielding effect”. The differences in elastic modulus between the bone and the metallic implant may lead to revision surgery [1,2,3,4,5,13,14]; thus, most research efforts have been centered on reducing the elastic modulus of the implant alloy. There have been some progress in this direction: while conventionally/commonly used α- or (α + β)-Ti alloys (including consecrated Ti-6Al-4V) exhibit a modulus of about 100–110 GPa, β-Ti alloys exhibit moduli of around 55–70 GPa [15,16,17,18,19,20], which is much closer to the elastic modulus of cortical bone (approximately 30 GPa) [21]. Associated with the Young’s modulus should be a sufficiently high yield strength, which, for the β-Ti alloy, seems to be quite a difficult requirement as the yield strength generally has medium values for the β-single microstructure [6].

Consequently, in terms of the requirements described above, the Ti–Nb–Zr–Ta system has proven to be very suitable, especially in terms of good biocompatibility and low Young’s modulus [22,23,24,25]. For the last few years, the Ti–Nb–Zr–Fe system has also been reported with good results [9,10,11,26,27,28,29], being a less expensive alternative. However, the ways/modalities to achieve the above-mentioned properties/requirements for these metastable β-Ti alloys are still being debated; there are three that have been the most exploited and explored.

One of them refers to finding the optimal chemical composition range of alloying elements that can satisfy the combination of characteristics: a high biocompatibility and β-stabilizing character, the capacity of decreasing the Young’s modulus with simultaneous increase of the yield strength, and the capacity to suppress the martensitic transformation (β→α″) by lowering the temperature Ms in order to obtain the single β-phase. Considering that many elements can determine only a gradual level of these characteristics, there is a need to find a proper balance/range of the envisaged alloying elements to obtain the best combination of alloy characteristics. From this perspective, there are some appreciated approaches that have been tried to optimize the composition based on electronic theory that use the Bo–Md diagram [30] and the simple calculation of the *e*/*a* ratio (valence electrons per atom) [31]. All these reported theoretical results/calculations have proven to be very useful for alloy design, but in general terms, for more exact tailoring/optimization of the necessary chemical compositions, the experimentally results of real alloys has been always more efficient/appreciated. Therefore, finding an optimal chemical composition that can accomplish the above enumerated requirements still represents a challenging experimental attempt.

The second modality explored to meet the above requirements for β-Ti alloys used for implants is to introduce oxygen alongside the alloying elements. Here, however, some uncertainties regarding the influence degree of two dichotomous manifestations of the oxygen still persists: on one hand, oxygen is considered a α-stabilizing element with respect to the β-transus temperature, which increases with oxygen addition [32]; on the other hand, the β-stabilizing nature of oxygen is more often taken into account and reported on considering its strong interstitial strengthening character [4,5,6] that can thus inhibit stress-induced martensitic transformation (β→α″) [33,34,35,36]. It seems that the first manifestation is less influential than the second one if a sufficiently high content of β stabilizing elements is taken into account, since the reports on the second direction of manifestation are more numerous [7,11,33,34,35,36,37,38].

The third tried modality of achieving suitable properties for metastable β-Ti alloys refers to thermo-mechanical processing of the alloy (i.e., the application of a severe plastic deformation (SPD) combined with a solution treatment (ST)). This approach seems to be very effective considering that, first of all, SPD is a powerful reducer of the grain size until ultrafine or even nanometer dimension [39,40,41]; by this, the Young’s modulus can strongly decrease with yield strength enhancing. Second, ST can keep the single β-phase if applied in the super β-transus variant [42]. From this perspective, there are some encouraging reports, but on particular alloy compositions. For example, for the Ti–Nb–Zr alloys combination [43] in the SPD + ST state (without expensive Ta), the modulus varies between 59–70 GPa for a Nb content around 35 wt.%. If adding Fe for the Ti–35Nb–10Ta–(Fe) alloy combination [44], the elastic modulus is about 75 GPa with a bending strength of 853 MPa. Referring to the role of Fe, early reports have shown that more than 2.5% Fe is discouraged due to detrimental mechanical performances [45,46] (too high strength, modulus, and fragility). Thus, it is considered now that Fe can be used as a minor alloying element (<2% wt.) [47], based on works [9,48] (Ti–Nb–Zr–Fe alloys) and [49] (Ti–Nb–Fe alloys) that report promising results. In 2009, [50] showed for the alloy Ti–28Nb–13Zr–0.5Fe wt.% (ST + water quenching-w.q.) a yield strength of 800 MPa, elongation of 13%, and an elastic modulus of 60 GPa with a microstructure composed of β-grains. In addition, Zr is an excellent ω-suppressor [47]. Concerning the Ti–Nb–(Ta)–Zr–Fe–O family alloys, with Fe and O additions, only a few reports [11,51,52,53] have shown that in the cold-rolling (CR) status or after a (CR and ST) combination, the Young’s modulus is about 60–90 GPa and the ultimate tensile strength-UTS between 900 and 1350 MPa, depending on the processing parameters.

Finally, there are some directions to follow to achieve the best requirements for the β-Ti alloys, but there are not enough explored all together, and their combination and influential degree are still raising problems for good optimization, with more or less success. A key issue is the selection of the technological parameters to be applied: the total deformation degree for SPD and the ST parameters (heating temperature, maintaining time, and quenching speed) on the one hand, and both coupled with sufficient high amount of β-stabilizing elements and an enough content of oxygen on the other hand. 

Consequently, the present paper proposes a β-Ti alloy of Ti–Nb–Zr–Fe–O type with the purpose to obtain a lower Young’s modulus by combining the above-mentioned possible modalities: (a) use of a large amount of β-stabilizing elements with high biocompatibility and Fe replacing Ta; (b) addition of oxygen; and (c) application of SPD and ST with optimized parameters. By this, the intention is to offer new data concerning the possibility of improving the mechanical properties for a promising particular composition of a β-Ti alloy intended to be used for hard tissue orthopedic implants.

## 2. Materials and Methods 

### 2.1. Synthesis and Thermomechanical Processing of the Studied Alloy

The obtained chemical composition for the studied Ti-Nb-Zr-Fe-O alloy (R&D company, Bucharest, Romania) is (wt.%): Ti—57.16%; Nb—34.18%; Zr—7.60%; Fe—0.90%; O—0.16%.

The synthesis of the alloy was based on the use of a levitation induction melting furnace FIVE CELES-MP25 (Five’s Group Company, Paris, France) with a nominal power of 25 kW and a melting capacity of 30 cm^3^ with a high vacuum of 10^−4^–10^−5^ mbar. During synthesis operation, an intensified stirring of the melted alloy was applied. To obtain a high chemical homogeneity, the re-melting of the ingots was performed twice.

**Figure 1:**
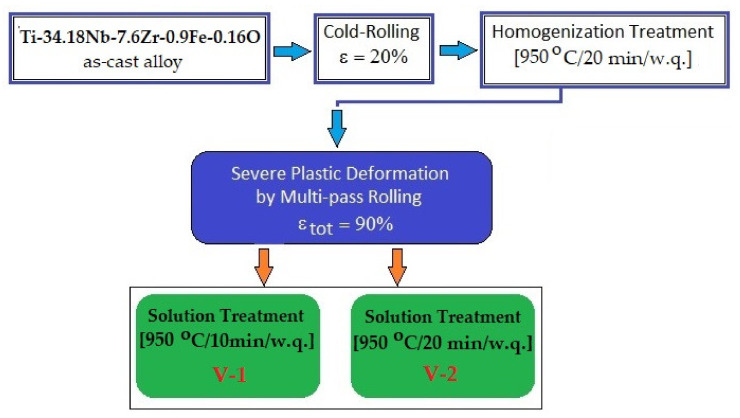
Integral schema for processing the studied Ti–Nb–Zr–Fe–O alloy.

To obtain a quality-homogenized precursor (Figure 1), the as-cast alloy was processed as follows: (a) cold rolling (CR) with a relative reduction of ε = 20% using a Mario di Maio LQR120AS rolling-mill (Mario di Maio Inc., Milano, Italy); the applied rolling speed was 3 m/min; no lubrication was required and an ultrasonic bath (CNCworld.ro, Arad, Romania) of ethylic alcohol (ChimReactiv S.R.L., Bucharest, Romania) at 60 °C was used to clean the as-cast alloy before CR; and (b) homogenization treatment (HT) performed above the β-transus temperature: 1223 K/950 °C/20 min/w.q.; for that, a GERO SR 100 × 500 type oven (Carbolite-Gero Inc., Neuhausen, Germany) was used under high vacuum.

The homogenized alloy was further thermomechanically processed by a combination of SPD using the multi-pass rolling-MPR (Mario di Maio Inc., Milano, Italy) method with two variants of ST. The MPR process was performed using 10 rolling passes with ε_tot_ = 90%. For both ST cases, the heating temperature and the cooling medium were similar (1223 K/950 °C and water, respectively), while the holding times were different: 10 min for variant 1 (V-1), and 20 min for variant 2 (V-2). Both STs were performed using same oven as for homogenization. 

### 2.2. Micro-Structural and Mechanical Analysis of the Studied Alloy 

For the cutting procedure of the alloy, necessary for obtaining the envisaged specimens, we used a Metkon MICRACUT 200 type machine (Metkon Instruments Inc., Bursa, Turkey) with diamond cutting disks. The resin of a Buehler Sampl-Kwick type (Buehler Company, Esslingen, Germany) was used to fix the specimens. A Metkon Digiprep ACCURA type machine (Metkon Instruments Inc., Bursa, Turkey) was used to abrade the samples with SiC paper-1200 grit (Struers Inc., Cleveland, OH, USA); the subsequent mechanical polishing was performed using a Buehler VibroMet2 machine (Buehler Ltd., Lake Bluff, IL, USA) with diamond pastes of 6, 3, and 1 μm successive parameters (DMT-Diamond Machine Technology Inc., Marlborough, MA, USA), and 0.03 μm colloidal silica (Struers Inc., Cleveland, OH, USA).

The microstructure of the alloy for all studied variants were analyzed using X-ray diffraction (XRD), scanning electron microscopy in back scattering electron mode (SEM-BSE), and transition electron microscopy (TEM).

Conventional X-ray diffraction was performed at room temperature using a Panalytical X’Pert PRO MRD diffractometer (Malvern Panalytical Ltd., Malvern, UK) with a Bragg-Brentano θ/θ geometry and a Cu K_α_ radiation source (λ = 0.15418 nm, 40 kV, 30 mA). The scanning interval was between 30–80° of the 2θ (°) with a step size of 0.02°. The rolling direction was set to be parallel to the direction of X-rays projected onto the sample surface. The recorded XRD patterns were fitted using the PeakFit software package (version 4.11, Systat Software Inc., London, UK). 

The SEM-BSE analysis was performed using a scanning electron microscope (TESCAN VEGA II—XMU type, Tescan Orsay Holding, a.s., Brno, Czech Republic). To observe the alloy texture and grain deformation evolution, the specimens in SPD state were analyzed in the RD-ND cross-section (RD—rolling direction; ND—normal direction).

The TEM analysis was performed using a transmission electron microscope, a TECNAI G2 F30 S-Twin HRTEM (FEI Company, Hillsboro, OR, USA).

The Gatan MicroTest-2000N-type machine (Gatan Inc., Pleasanton, CA, USA) with a strain rate of 1 × 10^−4^ s^−1^ was used to perform the tensile tests. The tensile test specimens had a “dog-bone” shape with a calibrated part as follows: 2 mm width, 0.8 mm thickness, and 10 mm gauge length. Based on the obtained data, the stress-strain curves for each processing variant were determined based on which the average values of the mechanical characteristics were also obtained: the ultimate tensile strength (σ_UTS_); yield strength (σ_0.2_); the elongation to fracture (ε_f_); the elastic modulus (E); and the elastic energy (We). For each mechanical characteristic, the standard deviation (SD) was also calculated.

## 3. Results and Discussions

### 3.1. The Reason for Selecting the Thermomechanical Processing Parameters

The β-titanium alloys are considered greatly workable in cold and annealed condition due to the presence of a single β-phase with bcc (body centered cubic) structure at ambient temperature. The studied alloy was a β-metastable alloy due to the used β-stabilizing alloying elements (Nb mainly, and Fe). The reason for applying the combination of the SPD and ST is to obtain an integral β-structure with improved mechanical properties that can lead to the use of the alloy as a biomaterial for orthopedic implants. For that purpose, it is necessary to apply the ST above the β-transus temperature (the so named “super transus solution treatment”) for a set time and rapid cooling of the sample until room temperature. The β-transus temperature (T_β_) represents an important parameter for selecting the heat treatment temperatures. The T_β_ value strongly depends on the alloying elements: α-stabilizers increase T_β_, β-stabilizers decrease T_β_, while the neutral elements do not change T_β_ [42]. Equation (1) to find the T_β_ value is given below [32]:

T_β_ = 882 + 21.1 × (Al%) − 9.5 × (Mo%) + 4.2 × (Sn%) − 6.9 × (Zr%) − 11.8 × (V%) − 12.1 × (Cr%) − 15.4 × (Fe%) + 23.3 × (Si%) + 123 × (O%) (°C) (1)

For the present studied alloy, the calculated T_β_ is:

T_β_ = 882 − 6.9 × (Zr%) − 15.4 × (Fe%) + 123 × (O%) = 882 − 6.9 × (7.60%) − 15.4 × (0.9%) + 123 × (0.16%) = 835.38 °C (2)

**Figure 2:**
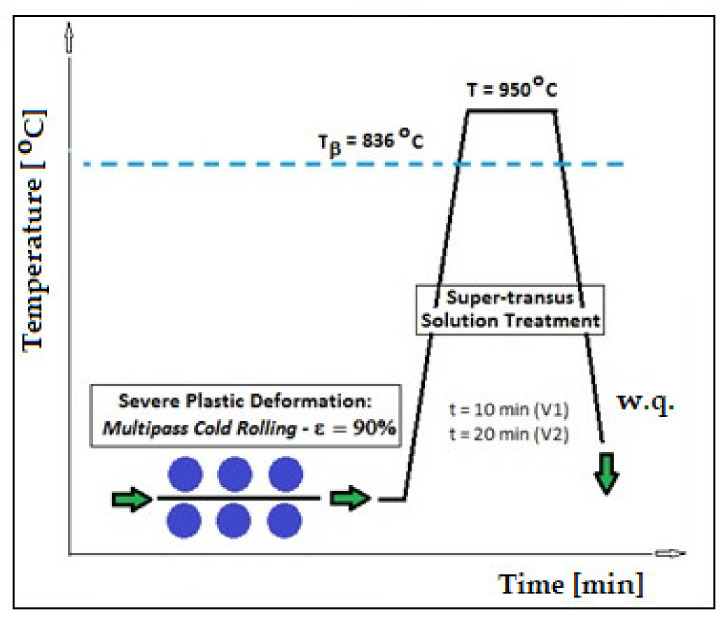
Schema for the thermomechanical processing of the studied Ti–Nb–Zr–Fe–O alloy by severe plastic deformation and solution treatment.

Figure 2 represents the schema of the applied SPD and ST processes for the studied alloy with established parameters for both V-1 and V-2 variants. According to [42], the super transus solution treatment for β-Ti alloys comprises a heating of the alloy with about 30–60 °C above T_β_ (for the present case it means of about 900 °C). Meanwhile, for similar alloys with almost the same amount of Nb, a ST above T_β_ has been successfully applied with 950 °C as the heating temperature, followed by quenching [12,54,55,56,57]. As a result, for the present study, we decided to also establish the ST temperature at 950 °C with a holding time in two variants: 10 min (V-1) and 20 min (V-2). The reason is that for a longer holding time, the risk/possibility arises of obtaining a large/coarse β-grain, a fact that would be detrimental for the final mechanical properties. The quenching medium was water to keep the single β-phase at room temperature. The reason is that the single β-phase facilitates the down-stream cold working operations [12], hence the metastable β-alloys were supplied in the ST-ed condition.

For the microstructural analysis, it will be also useful to find the temperature for starting the martensitic transformation (Ms), and to calculate the Mo_eq_ parameter, which represents a generally accepted parameter to characterize the stability of the β-phase for a disposed chemical composition [42,54].

The formula from [58] can be used especially for β-Ti alloys with high β stability to calculate the Ms. Thus, for the studied alloy, the calculated Ms (K) is:

Ms (K) = 1156 − 17.6 × (wt.% Nb) − 41.2 × (wt.% Zr) = 241.312 K = −31.688 °C = ~−32 °C(3)

Concerning the Mo_eq_ parameter as a measure of the β-stabilization level of the alloying elements, the equation for its determination, according to [12,59], is as follows:

Mo_eq_ = 1.0 × (wt.% Mo) + 0.67 × (wt.% V) + 0.44 × (wt.% W) + 0.28 × (wt.% Nb) + 0.22 × (wt.% Ta) + 2.9 × (wt.% Fe) + 1.6 × (wt.% Cr) − 1.0 × (wt.% Al) (4)

For the present case, the resulted Mo_eq_ is:

Mo_eq_. = 0.28 × (34.18% Nb) + 2.9 × (0.90% Fe) = 12.1804% (5)

Conforming to [12], β-Ti alloys with calculated Mo_eq_ between 10–30% are metastable and, in addition, heat treatable and deeply hardenable. Thus, the studied alloy with a Mo_eq_ of 12.18% seems to also be metastable, conforming to this calculation; following the next experimental analysis, this fact will be proven below. Thus, when characterizing/analyzing the alloy microstructure, the two parameters of Ms and Mo_eq_ will be considered as useful landmarks for future correlations with experimental data (i.e., the XRD analysis and also SEM/TEM imaging).

### 3.2. Micro-Structural Analysis of the Studied Alloy

**Figure 3:**
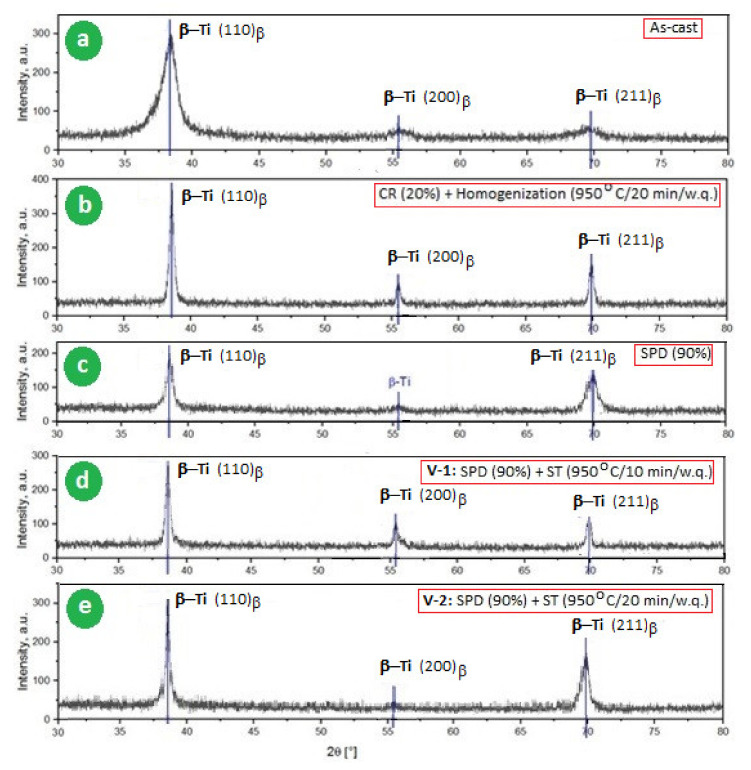
The XRD spectra of the studied alloy: (**a**) the as-cast sample; (**b**) after the homogenization treatment; (**c**) after the SPD (90%) processing; (**d**) V-1: SPD-MPR (ε_tot_ = 90%) + ST-1 (950 °C-10 min-w.q.); and (**e**) V-2: SPD-MPR (ε_tot_ = 90%) + ST-2 (950 °C-20 min-w.q.); ST-1: solution treatment-1; ST-2: solution treatment-2.

The microstructural analysis of the studied alloy was done for all of the experimental variants using XRD, SEM, and TEM analysis. Figure 3 provides the XRD spectra for all variants of the studied alloy. The main common denominator for all XRD variants was the presence of the peaks corresponding only to the β-phase. The intense (110)_β,_ (200)_β_, and (211)_β_ diffraction peaks of the β-phase with bcc structure, space group Im3m, were labelled according to ICDD no. 04-002-8708 [60].

Thus, based on Figure 3, it can be stated that XRD analysis demonstrates the β-stabilizing character of the alloying elements used (~42% in total, without oxygen), even after the application of SPD (Figure 3c) or ST (Figure 3d,e) during which there is a risk of secondary precipitation such as α, α′, ω, or α″-martensite, which may endanger the mechanical properties by increasing the fragility/resilience [61,62,63,64]. These phases are unfavorable to the processability of the material.

Possible precipitation of the orthorhombic α″ martensite or hexagonal α′ in the β matrix can considerably lower the modulus values and improve ductility, but with a corresponding strength decrease. The XRD spectra from Figure 3c did not reveal the presence of α″ diffraction peaks, meaning that stress-induced-martensitic (SIM) transformation does not occur due to high β phase stability. The (110)_β_ peak intensity for the SPD sample was 200 a.u. (Figure 3c), which is half of the value corresponding to the alloy sample in the homogenized state (Figure 3b). This is due to well-known phenomena—the crystal lattice distortion and the dislocation density increase during the SPD process. For the present case, it seems that the Mo_eq_ value is sufficiently high to ensure a high β-stabilizing character without the risk of forming α or α′, and the temperature Ms is sufficiently low to prevent the precipitation of α″ martensite during SPD by SIM transformation.

In contrast to the influence of α″ precipitation, the possible formation of the athermal ω phase during ST is correlated with a rise in strength, reduced ductility, and, in most cases, an unsatisfactory increase in elastic modulus [42]. However, knowing that the addition of about 5–7 wt.% of Zr to Ti–Nb/Mo–Fe alloys represents a sufficient quantity to suppress the formation of athermal ω precipitates during ST (water quenching) [47], it can be concluded that the Zr content (7.6 wt.%) from the present case, alongside other alloying elements, would be sufficient to ensure this desideratum. This finding corroborates all of the above presented results.

**Figure 4:**
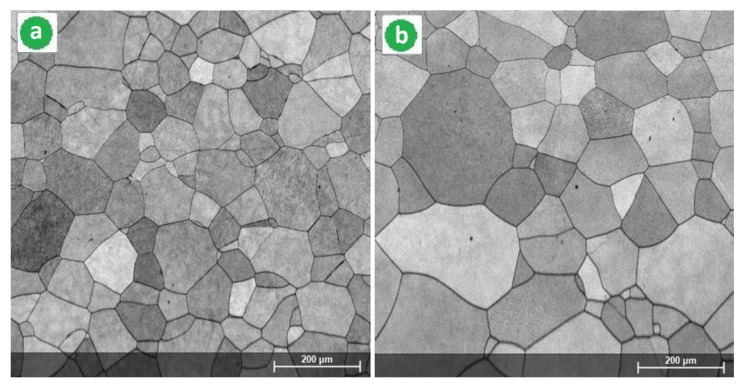
SEM-BSE images of the Ti-34.18Nb-7.6Zr-0.9Fe-0.16O alloy microstructures in the as-cast state (**a**) and after the homogenization treatment (**b**).

**Figure 5:**
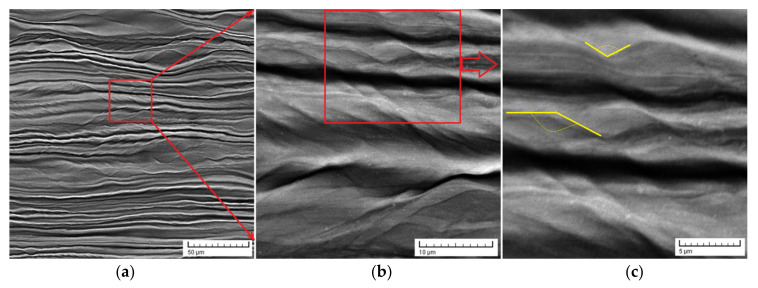
SEM-BSE images of the sample processed by SPD-MPR (ε_tot_ = 90%); (**a**)–(**c**)—represent the same sample with increasing magnification of the visual field sequences from (**a**) to (**c**); yellow mark—shear bands with large misorientation angles.

**Figure 6:**
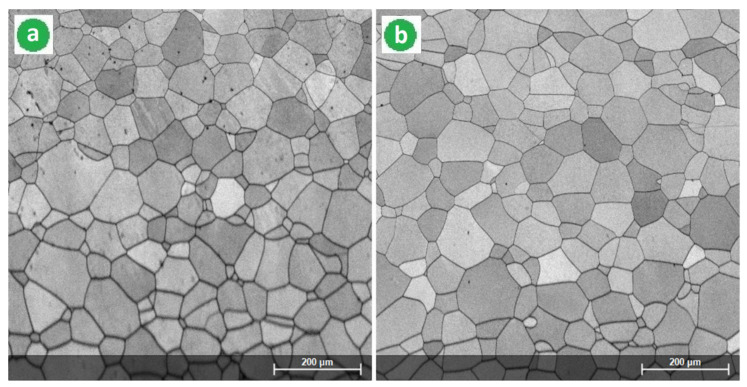
SEM-BSE images corresponding to the studied alloy after SPD and ST: (**a**) V-1: MPR (ε_tot_ = 90%) + ST1 (950 °C-10 min-w.q.); (**b**) V-2: MPR (ε_tot_ = 90%) + ST2 (950 °C-20 min-w.q.).

Continuing the microstructural analysis with SEM images, Figure 4 corresponds to the alloy in the cast and homogenized state, respectively. Figure 5 corresponds to the alloy processed by SPD (90%) and Figure 6 to the alloy processed by the combination [SPD + ST] with two variants V-1 and V- 2. Figure 4 proves once again that the alloy consists only of the β-phase due to the structure formed only from equiaxed grains of the β-phase. The determined average dimensions of the β grains were 122 μm for the as-cast specimen, and largest for the specimen in the homogenized state of 148 μm due to the grain expanding during the homogenization treatment. It should be noted that other scientific reports have also indicated the single β-phase for almost comparable chemical content (high β-stabilizing elements) and similar thermomechanical processing [12,34,37,44,61,62].

After the homogenization treatment, the studied alloy was processed by SPD-MPR with a total deformation degree of ε_tot_ = 90%. The SEM images of the obtained microstructures correspond to Figure 5, which indicates a strong texture on the rolling direction β grains. For the present case, as a result of a very high degree of deformation, the observed deformation products seemed to be twin bands or shear bands with large misorientation angles, marked with yellow in Figure 5c. It is well-known that the SPD process with high deformation degree, as here, can induce/provoke the apparition of the stress-induced martensite α″. However, this phenomenon does not occur here, proven by the above XRD spectra. Additionally, it is well-known that the twin/shear bands can help to further fragment and refine the grain during the process of SPD [61,62]. Indeed, the XRD spectra for the sample processed by SPD had much smaller diffraction peaks compared to the precursor homogenized sample, proving by this a serious diminution of the grain dimension. 

After SPD processing, both samples were subjected to two variants of ST (V-1 and V-2) with the same heating temperature (950 °C) and quenching medium, with 10 min as holding time for V-1 and 20 min for V-2. Figure 6 shows the SEM images of the resulting microstructures formed only from the equiaxed single β-phase. The average dimension of the grain size was measured using the intercept method and were ~72 μm for V-1 and ~94 μm for V-2. The selected ST parameters for both V-1 and V-2 variants led to the entire recrystallization of the β-phase from SPD status. Comparing both images from Figure 6 in terms of grain size, it appears that the extra 10 min for the corresponding ST holding time with variant V-2 versus V-1 produced an increase with about 23% of the β grain dimension; In addition, the grains were more dimensionally homogeneous. Furthermore, the obtained β grains were small in size, which represent a good premise to obtain a higher strength compared to the homogeneous state; this aspect will be analyzed in the following. Even if the holding time is longer than 20 min, the premise of obtaining a single β-phase remains if considering a super-transus ST with water quenching and a high amount of β-stabilizing elements.

**Figure 7:**
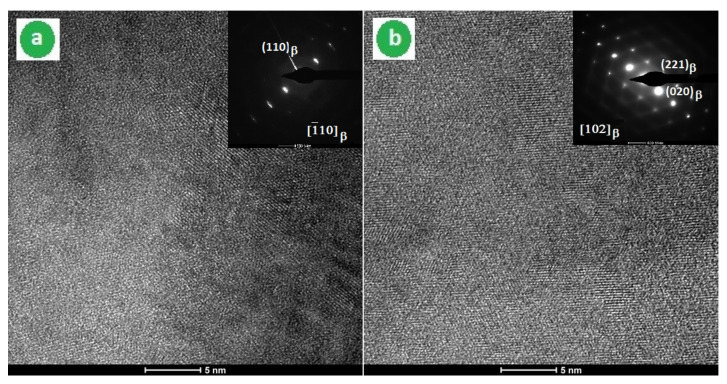
HRTEM images corresponding to both ST variants—V-1 and V-2—applied on the studied alloy: (**a**) V-1: bright field image and the SAED image observed along the [110]_β_ zone axis; (**b**) V-2: bright field image and the SAED image observed along the [102]_β_ zone axis.

To verify the formation of a single β-phase detected by XRD spectra and visible in SEM images, a HRTEM study was carried out on samples corresponding to V-1 and V-2 variants of the applied ST. Figure 7 shows the bright field image and the corresponding selected-area electron diffraction (SAED) patterns. The bright field images corresponding to both ST variants were clean of precipitates and the SAED patterns showed distinct spots of the β-phase only.

### 3.3. Mechanical Properties Analysis of the Studied Alloy after SPD and ST Processing 

**Figure 8:**
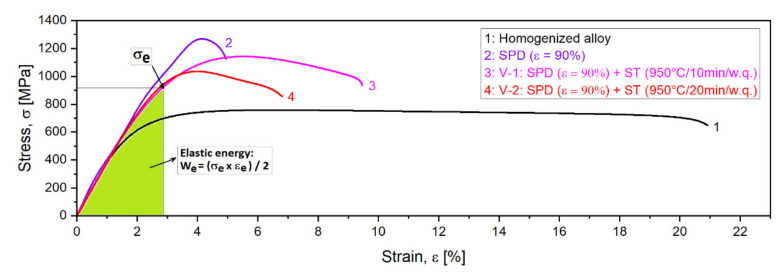
The stress-strain curves of the samples processed by SPD and ST compared to the initial homogenized state.

To evaluate the mechanical properties, tensile tests were performed for the V-1 and V-2 samples. The resulted strain-stress curves are indicated in Figure 8. Table 1 shows the average values for the ultimate tensile strength (UTS); yield strength (YS); elongation to fracture (ε_f_); elastic modulus (E); and elastic energy (We).

**Figure 9:**
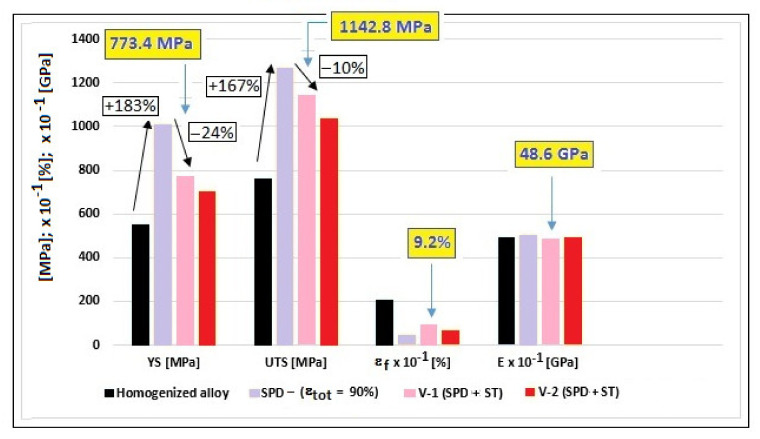
Mechanical properties of the studied alloy for all experimented states; in yellow squares—the promising combination of mechanical properties corresponding to the V-1 processing variant.

In Table 1, the values of the Young’s modulus, around 48–50 GPa, show an encouraging level for all states of the experimented alloy. With regard to the YS and UTS values, starting with the homogenized state for which the UTS and YS values were at a medium level, they increased significantly for the SPD/MPR state (the values almost double) and remained at a high level after the ST (V-1 and V-2) (Figure 9), with only a small decrease. Figure 9 also indicates the good combination of the obtained mechanical properties for the V-1 variant of SPD + ST, with the lowest modulus of 48.6 GPa.

The GATAN MicroTest-2000N-type machine used for the present experiments was not equipped with an external extensometer. The machine measures the sample elongation as grips position displacement. The evaluation of the Young’s modulus was performed based on the displacement data measured as a function of grips position. Considering that the displacement measurements can take into account the compliance/stiffness of the machine and gripping technique in addition to the sample deformation, the results for the Young’s modulus values may be slightly lowered in some cases. Therefore, a future verification/assessment of the present results using complementary techniques can be important. However, it should be emphasized that in this case, the stiffness could only be high because the loads were very small; also, the measurement accuracy was less than 1 N.

For the studied alloy, the elastic energy (We) is a very important parameter as this biomaterial is usually used for orthopedic implants in the range of elastic deformation [56]. In the case of hyper-elastic alloys, the stress-induced martensite transformation can be displayed in the stress-strain curve; therefore, it is more reasonable to calculate the cyclic dissipation energy under cyclic loading than the elastic energy [56,65,66]. However, for the studied alloy, which did not present the stress platform of martensite transition due to very low Ms (Ms = −32 °C), the calculated elastic energy (We) is an important parameter for analyzing the capabilities to work in the range of elastic deformation. The elastic energy (We) can be assessed using Figure 8 by applying Equation (6) from [56]:

We = ½ (σ_e_ × ε_e_) (6)

where σ_e_ and ε_e_ are the values from stress-strain curve (Figure 8).

The calculated We values for the studied alloy after two variants of SPD + ST are listed in Table 1. The elastic energy for V-1 was 11.3 × 10^6^ J/m^3^ and 9.8 × 10^6^ J/m^3^ for V-2. Considering that both values considerably exceeded the value corresponding to Ti-6Al-4V alloy as reported as 2.8 × 10^6^ J/m^3^ in [56], it can be concluded that the studied alloy can be a good candidate for a new biomaterial.

**Figure 10:**
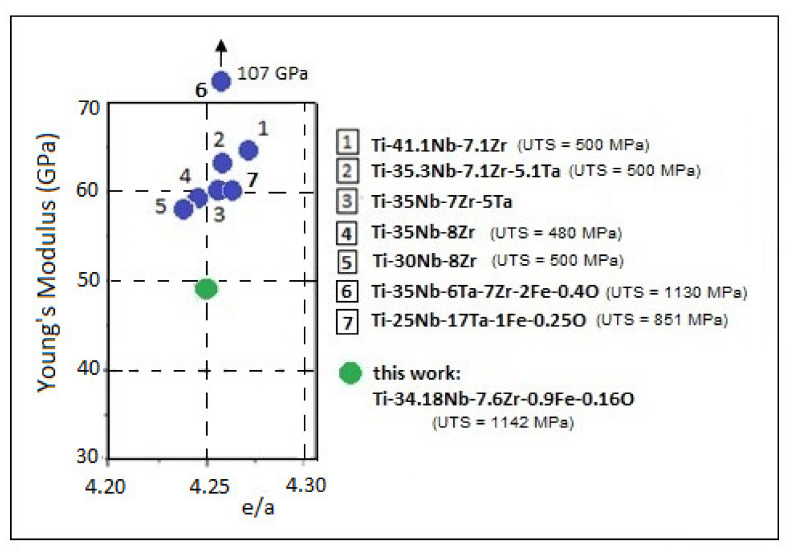
The Young’s modulus of the studied alloy compared to that of other similar β-Ti alloys. Alloy (1) from [70]; Alloy (2) from [24]; Alloy (3) from [71]; Alloy (4) and (5) from [43]; Alloy (6) from [11]; Alloy (7) from [66].

Concerning the key mechanical parameter for the β-Ti alloys—the Young’s modulus—there are several studies [25,67] that indicate *e*/*a* (valence electrons per atom) as a dominant factor governing the modulus of bcc transition metals including β-Ti alloys. It has been reported that if *e*/*a* decreases, the shear modulus c’ = (c11−c12)/2 and bulk modulus B of a bcc crystal also decrease, causing the β phase to become unstable [25,67,68,69,70,71]. Consequently, if β-stabilizing alloying elements increase, the *e*/*a* also increases, meaning a strong stabilization of the β-phase. Conforming to [72], if the values for the *e*/*a* ratio are between 4.24 and 4.26, a minimum modulus can be obtained for the β-Ti alloys. For the present case, the value of 4.253 for *e*/*a* falls within the reported optimal range. Therefore, the high level of the β-stabilizing elements (~42%) correlated with *e*/*a* value represent a good premise to expect a low level of the modulus, as demonstrated by the obtained values. In addition, knowing that a correct appreciation is based on a comparison, Figure 10 compares the Young’s modulus value of the studied alloy (48.6 GPa for V1 variant) to that of other similar β-Ti alloys with close chemical composition and almost similar *e*/*a* values (4.24–4.26). By this, the obtained value can be highlighted correctly as a very promising one. Next to each alloy is indicated the UTS value, which is also an important indicator along with the Young’s modulus. A significant increase of this value can be observed with the addition of oxygen in the composition.

Another important observation/comment derives from [43], according to which the Young’s modulus decreases with increasing Zr content and, at a certain Nb content, reaches a minimum value, even if it otherwise increases slowly with increasing Nb content. This sensitivity of the Zr versus Nb content that can influence the obtaining of a suitable modulus leads to the conclusion that the Zr/Nb compositional ratio must be conjugated/adapted very carefully in order to obtain the lowest possible value of the modulus. If considering a range of 0.17–1.2 for the Zr/Nb ratio explored by [43] with encouraging results, it can be presumed that the ratio of 0.22 for the here studied alloy could represent a satisfied premise for a suitable low modulus. In addition, [73] also reported a low Young’s modulus for a Zr/Nb ratio of 1.0, for the Ti-13Nb-13Zr alloy. Thus, the range for the Zr/Nb ratio also seems to have been considered by other studies. With regard to the justification of Fe addition alongside with Nb and Zr, [8] reports that Fe of approximately 2 wt.% increased the strength considerably, preserving both the ductility and Young’s modulus at minor levels. Therefore, if we correlate these data with the Fe content used for the present alloy (0.90 wt.%) and with the obtained modulus value, it can be presumed that the Fe content also helps with these encouraging results. 

Concerning oxygen, many reports referring to oxygen-containing Ti–Nb–Zr–Ta alloys indicate a strong strengthening character of the interstitial oxygen, capable of increasing the resistance of the β matrix and partially suppressing the (β→α″) transformation [74,75,76]. Furthermore, it has been reported that Ms-temperature indicates a loss of approximately 160 K if adding 1 at.% O in the Ti-22Nb alloy [77]. Even if oxygen increases the T_β_ temperature (conforming to Equation (2)) but considering the low Ms-temperature (calculated at −32 °C), it can be presumed that the conditions to increase the resistance of the β-phase and thus to decrease the modulus are very promising.

## 4. Conclusions

Two variants of ST combined with SPD were applied for a Ti–Nb–Zr–Fe–O alloy with the intention of obtaining a low elastic modulus with sufficient high strength. The total reduction of SPD was ε_tot_ = 90% and the selected parameters for ST corresponded to a super-transus solution treatment.

The microstructure analyses provided by XRD, SEM, and HRTEM investigations revealed the presence of a single β-phase, proving by this that the amount and the type of β-stabilizing elements were sufficient enough to obtain the stable β-phase necessary for this type of alloy to be used in the range of elastic deformation for orthopedic implants.

The analyzed mechanical properties revealed promising values for YS and UTS of about 770 and 1140 MPa, respectively, with a low value for the Young’s modulus of 48.6 GPa. The conclusion is that satisfactory mechanical properties for this type of alloy can be obtained by optimizing the combination of SPD + ST parameters and the content of alloying elements, especially the Zr/Nb ratio.

## Figures and Tables

**Table 1:** Mechanical properties of the studied alloy: ultimate tensile strength (UTS); yield strength (YS); elongation to fracture (ε_f_); elastic modulus (E); elastic energy (We); SD (standard deviation).

The Alloy State	Mechanical Properties (SD-Standard Deviation)				
	YS (MPa)	UTS (MPa)	ε_f_ (%)	E (GPa)	We (J/m^3^)
Homogenized alloy	552.1 (10.8)	760.2 (13.3)	20.6 (0.6)	49.2 (1.6)	4.3 × 10^6^
MPR—(ε_tot_ = 90%)	1011.8 (11.3)	1268.6 (16.3)	4.8 (0.1)	50.3 (1.2)	16.6 × 10^6^
V-1: (ε_tot_ = 90%) + (950 °C-10 min-w.q.)	773.4 (11.2)	1142.8 (16.1)	9.2 (0.3)	48.6 (0.8)	11.3 × 10^6^
V-2: (ε_tot_ = 90%) + (950 °C-20 min-w.q.)	702.3 (10.2)	1036.4 (15.8)	6.8 (0.2)	49.3 (1.1)	9.8 × 10^6^

## Data Availability

The data presented in this study are available on request from the corresponding author.

## References

[1] Kaur M., Singh K. (2019). Review on titanium and titanium based alloys as biomaterials for orthopaedic applications. Mater. Sci. Eng. C.

[2] Bahl S., Suwas S., Chatterjee K. (2020). Comprehensive review on alloy design, processing, and performance of β Titanium alloys as biomedical materials. Int. Mater. Rev..

[3] Çaha I., Alves A.C., Rocha L.A., Toptan F. (2020). A Review on Bio-functionalization of β-Ti Alloys. J. Bio. Tribo. Corros..

[4] Valiev R.Z., Prokofiev E.A., Kazarinov N.A., Raab G.I., Minasov T.B., Strasky J. (2020). Developing Nanostructured Ti Alloys for Innovative Implantable Medical Devices. Materials.

[5] Froes F., Qian M. (2018). Titanium in Medical and Dental Applications.

[6] Kozlík J., Preisler D., Stráský J., Veselý J., Veverková A., Chráska T., Janeček M. (2021). Phase transformations in a heterogeneous Ti-xNb-7Zr-0.8O alloy prepared by a field-assisted sintering technique. Mater. Des..

[7] Zhang L.C., Chen L.Y. (2019). A review on biomedical titanium alloys: Recent Progress and Prospect. Adv. Eng. Mater..

[8] Kopova I., Strasky J., Harcuba P., Landa M., Janecek M., Bacakova L. (2016). Newly developed Ti–Nb–Zr–Ta–Si–Fe biomedical beta titanium alloys with increased strength and enhanced biocompatibility. Mater. Sci. Eng. C.

[9] Dal Bo M.R., Salvador C.A.F., Mello M.G., Lima D.D., Faria G.A., Ramirez A.J., Caram R. (2018). The effect of Zr and Sn additions on the microstructure of Ti-Nb-Fe gum metals with high elastic admissible strain. Mater. Des..

[10] Xu Y., Gao J., Huang Y., Rainforth W.M. (2020). A low-cost metastable beta Ti alloy with high elastic admissible strain and enhanced ductility for orthopaedic application. J. All. Comp..

[11] Strasky J., Harcuba P., Vaclavova K., Horvath K., Landa M., Srba O., Janecek M. (2017). Increasing strength of a biomedical Ti-Nb-Ta-Zr alloy by alloying with Fe, Si and O. J. Mech. Behav. Biomed. Mat..

[12] Kolli R., Devaraj A. (2018). A review of metastable beta titanium alloys. Metals.

[13] Okazaki Y., Katsuda S.-i. (2021). Biological Safety Evaluation and Surface Modification of Biocompatible Ti–15Zr–4Nb Alloy. Materials.

[14] Gurel S., Yagci M.B., Canadinc D., Gerstein G., Bal B., Maier H.J. (2020). Fracture behavior of novel biomedical Ti-based high entropy alloys under impact loading. Mater. Sci. Eng. A.

[15] Gepreel M.A.-H., Niinomi M. (2013). Biocompatibility of Ti-alloys for long-term implantation. J. Mech. Behav. Biomed. Mater..

[16] Ozan S., Lin J., Zhang Y., Li Y., Wen C. (2020). Cold rolling deformation and annealing behavior of a β-type Ti–34Nb–25Zr titanium alloy for biomedical applications. J. Mater. Res. Technol..

[17] Gupta A., Khatirkar R.K., Kumar A., Parihar M.S. (2018). Investigations on the effect of heating temperature and cooling rate on evolution of microstructure in an α + β titanium alloy. Mater. Res. Soc..

[18] Padmalatha T.S.R.V., Chakkingal U. (2019). The effect of heat treatment and the volume fraction of the alpha phase on the workability of Ti-5Al-5Mo-5V-3Cr alloy. J. Mater. Eng. Perform..

[19] Xu P., Zhou L., Han M., Wei Z., Liang Y. (2020). Flash-butt welded Ti6242 joints preserved base-material strength and ductility. Mater. Sci. Eng. A.

[20] Hanada S., Masahashi N., Semboshi S., Jung T.K. (2021). Low Young’s modulus of cold groove-rolled β Ti–Nb–Sn alloys for orthopedic applications. Mater. Sci. Eng. A.

[21] Niinomi M., Yi L., Nakai M., Liu H., Hua L. (2016). Biomedical titanium alloys with Young’s moduli close to that of cortical bone. Regener. Biomater..

[22] Haftlang F., Zarei-Hanzaki A., Abedi H.R., Kalaei M.A., Nemecek J., Málek J. (2019). Room-temperature micro and macro mechanical properties of the metastable Ti–29Nb–14Ta–4.5Zr alloy holding nano-sized precipitates. Mater. Sci. Eng A.

[23] Baltatu M.S., Vizureanu P., Geanta V., Tugui C.A., Voiculescu I. (2019). Mechanical tests for Ti-based alloys as new medical materials. IOP Publ. Conf. Ser. Mater. Sci. Eng..

[24] Elias L.M., Schneider S.G., Schneider S., Silva H.M., Malvisi F. (2006). Microstructural and mechanical characterization of biomedical Ti–Nb–Zr(–Ta) alloys. Mater. Sci. Eng. A.

[25] Tane M., Akita S., Nakano T., Hagihara K., Umakoshi Y., Niinomi M., Mori H., Nakajima H. (2010). Low Young’s modulus of Ti–Nb–Ta–Zr alloys caused by softening in shear moduli c0 and c44 near lower limit of body- centered cubic phase stability. Acta Mater..

[26] Fu Y., Wang J., Xiao W., Zhao X., Ma C. (2020). Microstructure evolution and mechanical properties of Ti–8Nb–2Fe-0.2O alloy with high elastic admissible strain for orthopedic implant applications. Prog. Nat. Sci. Mater. Int..

[27] Biesiekierski A., Lin J., Li Y., Ping D., Yamabe-Mitarai Y., Wen C. (2016). Investigations into Ti-(Nb,Ta)-Fe Alloys for Biomedical Applications. Acta Biomater..

[28] Li Q., Miao P., Li J., He M., Nakai M., Niinomi M., Chiba A., Nakano T., Liu X., Zhou K. (2019). Effect of Nb Content on Microstructures and Mechanical Properties of Ti-xNb-2Fe Alloys. J. Mater. Eng. Perform..

[29] Ehtemam -H.S., Attar H., Okulov I.V., Dargusch M.S., Kent D. (2021). Microstructural evolution and mechanical properties of bulk and porous low-cost Ti–Mo–Fe alloys produced by powder metallurgy. J. All. Comp..

[30] Abdel-Hady M., Hinoshita K., Morinaga M. (2006). General approach to phase stability and elastic properties of β-type Ti-alloys using electronic parameters. Scr. Mater..

[31] Abdel-Hady M., Fuwa H., Hinoshita K., Kimura H., Shinzato Y., Morinaga M. (2007). Phase stability change with Zr content in β-type Ti–Nb alloys. Scr. Mater..

[32] Naresh Kumar K., Muneshwar P., Singh S.K., Jha A.K., Pant B. (2015). Thermomechanical working and heat treatment studies on meta-stable beta titanium alloy (Ti15V3Al3Sn3Cr) plates. Mater. Sci. Forum.

[33] Wu H., Zhou J. (2018). The quantitative understanding on the influence of α″ phase on mechanical behavior of Ti-Nb-Ta-Zr-O alloy. J. All. Comp..

[34] Acharya S., Panicker A.G., Laxmi D.V., Suwas S., Chatterjee K. (2019). Study of the influence of Zr on the mechanical properties and functional response of Ti-Nb-Ta-Zr-O alloy for orthopedic applications. Mater. Des..

[35] Yokota K., Bahador A., Shitara K., Umeda J., Kondoh K. (2021). Mechanisms of tensile strengthening and oxygen solid solution in single β-phase Ti-35 at.% Ta + O alloys. Mater. Sci. Eng. A.

[36] Sochacka P., Jurczyk M.U., Kowalski K., Wirstlein P.K., Jurczyk M. (2021). Ultrafine-Grained Ti-31Mo-Type Composites with HA and Ag, Ta_2_O_5_ or CeO_2_ Addition for Implant Applications. Materials.

[37] Stráský J., Janeček M., Harcuba P., Preisler D., Landa M., Froes F.H., Qian M. (2018). 4.2-biocompatible beta-Ti alloys with enhanced strength due to increased oxygen content. Titanium and Medical Dental Applications.

[38] Preisler D. (2018). Oxygen-Strengthened Biomedical Beta Titanium Alloys. Master’s Thesis.

[39] Mohammed M.T., Khan Z.A., Siddiquee A.N. (2014). Beta Titanium Alloys: The Lowest Elastic Modulus for Biomedical Applications: A Review. World Acad. Sci. Eng. Technol. Int. J. Chem. Mol. Nucl. Mater. Metal. Eng..

[40] Zafari A., Ding Y., Cui J., Xia K. (2016). Achieving fine beta grain structure in a metastable beta titanium alloy through multiple forging-annealing cycles. Metal. Mater. Trans. A.

[41] Hsu H.C., Wu S.C., Kuo J.H., Ho W.F. (2021). Effects of Heat Treatments on the Structure and Mechanical Properties of Ti-25Nb-8Sn Alloy. J. Mater. Eng Perform..

[42] Soundararajan S.R., Vishnu J., Manivasagam G., Muktinutalapati N.R. (2020). Heat Treatment of Metastable Beta Titanium Alloys. IntechOpen.

[43] Kim K.M., Kim H.Y., Shuichi Miyazaki S. (2020). Effect of Zr Content on Phase Stability, Deformation Behavior, and Young’s Modulus in Ti–Nb–Zr Alloys. Materials.

[44] Amigó A., Vicente A., Afonso C.R.M., Amigó V. (2019). Mechanical properties and the microstructure of β Ti-35Nb-10Ta-xFe alloys obtained by powder metallurgy for biomedical applications. Metals.

[45] Lee C.M., Ho W.F., Ju C.P., Chern Lin J.H. (2002). Structure and properties of Titanium-25 Niobium-x iron alloys. J. Mater. Sci. Mater. Med..

[46] Hsu H.-C., Hsu S.-K., Wu S.-C., Lee C.J., Ho W.F. (2010). Structure and mechanical properties of as-cast Ti–5Nb–xFe alloys. Mater. Charact..

[47] Salvador Camilo A.F., Roveri-Dal Bo M., Lima D.D., Miranda Caetano R., Caram R. (2021). Experimental and computational investigation of Ti-Nb-Fe-Zr alloys with limited Fe contents for biomedical applications. J. Mater. Sci. Met. Corros..

[48] Xue P., Li Y., Li K., Zhang D., Zhou C. (2015). Superelasticity, corrosion resistance and biocompatibility of the Ti-19Zr-10Nb-1Fe alloy. Mater. Sci. Eng. C.

[49] Lopes E.S.N., Salvador C.A.F., Andrade D.R., Cremasco A., Campo K.N., Caram R. (2016). Microstructure, mechanical properties, and electrochemical behavior of Ti-Nb-Fe alloys applied as biomaterials. Metall. Mater. Trans. A.

[50] Cuil W.F., Guo A.H. (2009). Microstructures and properties of biomedical TiNbZrFe β-titanium alloy under aging conditions. Mater. Sci. Eng. A.

[51] Nocivin A., Cojocaru V.D., Raducanu D., Cinca I., Angelescu M.L., Dan I., Serban N., Cojocaru M. (2017). Finding an Optimal Thermo-Mechanical Processing Scheme for a Gum-type Ti-Nb-Zr-Fe-O Alloy. J. Mater. Eng. Perform..

[52] Furuta T., Kuramoto S., Hwang J., Nishino K., Saito T., Niinomi M. (2007). Mechanical Properties and Phase Stability of Ti-Nb-Ta-Zr-O Alloys. Mater. Trans..

[53] Wei Q., Wang L., Fu Y., Qin J., Lu W., Zhang D. (2011). Influence of oxygen content on microstructure and mechanical properties of Ti–Nb–Ta–Zr alloy. Mater. Des..

[54] Mohan P., Rajak D.K., Pruncu I.C., Behera A., Amigó-Borrás V., Elshalakany A.B. (2020). Influence of β-phase stability in elemental blended Ti-Mo and Ti-Mo-Zr alloys. Micron.

[55] Besse M., Castany P., Gloriant T. (2011). Mechanisms of deformation in gum metal TNTZ-O and TNTZ titanium alloys: A comparative study on the oxygen influence. Acta Mater..

[56] Li P., Ma X., Wang D., Zhang H. (2019). Microstructural and Mechanical Properties of β-Type Ti–Nb–Sn Biomedical Alloys with Low Elastic Modulus. Metals.

[57] Bahl S., Shreyas P., Trishul M.A., Suwas S., Chatterjee K. (2015). Enhancing the mechanical and biological performance of a metallic biomaterial for orthopedic applications through changes in the surface oxide layer by nanocrystalline surface modification. Nanoscale.

[58] Neelakantan S., Rivera-Diaz-del-Castillo R., Van der Zwaag S. (2009). Prediction of the martensite start temperature for β titanium alloys as a function of composition. Scripta Mater..

[59] Santhosh R., Geetha M., Nageswara Rao M. (2016). Recent developments in heat treatment of Beta titanium alloys for aerospace applications. Trans. Indian Inst. Met..

[60] Cullity B.D. (2001). Stock, Elements of X-ray Diffraction.

[61] Ozan S., Lin J., Li Y., Zhang Y., Munir K., Jiang H., Wen C. (2018). Deformation mechanism and mechanical properties of a thermo-mechanically processed β Ti–28Nb–35.4Zr alloy. J. Mech. Behav. Biomed. Mater..

[62] Ozan S., Li Y.C., Lin J.X., Zhang Y.W., Jiang H.W., Wen C.E. (2018). Microstructural evolution and its influence on the mechanical properties of a thermo-mechanically processed beta Ti-32Zr-30Nb alloy. Mater. Sci. Eng. A.

[63] Tang B., Chu Y., Zhang M., Meng C., Fan J., Kou H., Li J. (2020). The ω phase transformation during the low temperature aging and low rate heating process of metastable β titanium alloys. Mater. Chem. Phys..

[64] Ali T., Wang L., Cheng X., Liu A., Xu X. (2019). Omega phase formation and deformation mechanism in heat treated Ti-5553 alloy under high strain rate compression. Mater. Lett..

[65] Cui B., Yao J., Wu Y., Zhang X., Wang F.L., Sui J.H., Cai W. (2018). Precipitation behavior and mechanical properties of Ti–Ni–Nb–Co alloys. Intermetallics.

[66] Hussein A.H., Gepreel M.A.H., Gouda M.K., Hefnawy A.M., Kandil S.H. (2016). Biocompatibility of new Ti–Nb–Ta base alloys. Mater. Sci. Eng. C.

[67] Hao Y.L., Li S.J., Sun S.Y., Zheng C.Y., Yang R. (2007). Elastic deformation behavior of Ti–24Nb–4Zr–7.9Sn for biomedical applications. Acta Biomater..

[68] You L., Song X. (2012). A study of low Young’s modulus Ti-Nb-Zr alloys using d electrons alloy theory. Scr. Mater..

[69] Wang X., Zhang L., Guo Z., Jiang Y., Tao X., Liu L. (2016). Study of low-modulus biomedical β Ti–Nb–Zr alloys based on single-crystal elastic constants modeling. J. Mech. Behav. Biomed. Mater..

[70] Schneider S., Schneider S.G., Silva H.M., Neto C.M. (2005). Study of the non-linear stress–strain behavior in Ti–Nb–Zr alloys. Mater. Res..

[71] Raabe D., Sander B., Friak M., Ma D., Neugebauer J. (2007). Theory-guided bottom-up design of β-Ti alloys as biomaterials based on first principles calculations: Theory and experiments. Acta Mater..

[72] Todd R., Armstrong D. (2006). Gum metal and related alloys. Encyclopedia of Materials: Science and Technology.

[73] Lee T., Lee S., Kim I.S., Moon Y.H., Kim H.S., Park C.H. (2020). Breaking the limit of Young’s modulus in low-cost Ti–Nb–Zr alloy for biomedical implant applications. J. Alloys Comp..

[74] Wei L.S., Kim H.Y., Miyazaki S. (2015). Effects of oxygen concentration and phase stability on nano-domain structure and thermal expansion behavior of Ti–Nb–Zr–Ta–O alloys. Acta Mater..

[75] Nakai M., Niinomi M., Akahori T., Tsutsumi H., Ogawa M. (2009). Effect of Oxygen Content on Microstructure and Mechanical Properties of Biomedical Ti-29Nb-13Ta-4.6Zr Alloy under Solutionized and Aged Conditions. Mater. Trans..

[76] Niinomi M., Nakai M., Hendrickson M., Nandwana P., Alam T., Choudhuri D., Banerjee R. (2016). Influence of oxygen on omega phase stability in the Ti-29Nb-13Ta-4.6Zr alloy. Scr. Mater..

[77] Kim J.I., Kim H.Y., Hosoda H., Miyazaki S. (2005). Shape Memory Behavior of Ti-22Nb-(0.5–2.0)O(at%). Mater. Trans..

